# Gastroprotective effects of Kangfuxin against water-immersion and restraint stress-induced gastric ulcer in rats: roles of antioxidation, anti-inflammation, and pro-survival

**DOI:** 10.1080/13880209.2019.1682620

**Published:** 2019-11-07

**Authors:** Shan Lu, Daoshun Wu, Guibo Sun, Funeng Geng, Yongmei Shen, Jin Tan, Xiaobo Sun, Yun Luo

**Affiliations:** a Key Laboratory of Bioactive Substances and Resources Utilization of Chinese Herbal Medicine, Ministry of Education, Institute of Medicinal Plant Development, Peking Union Medical College and Chinese Academy of Medical Sciences, Beijing, China;; b Beijing Key Laboratory of Innovative Drug Discovery of Traditional Chinese Medicine (Natural Medicine) and Translational Medicine, Institute of Medicinal Plant Development, Peking Union Medical College and Chinese Academy of Medical Sciences, Beijing, China;; c Key Laboratory of New Drug Discovery Based on Classic Chinese Medicine Prescription, Chinese Academy of Medical Sciences, Beijing, China;; d Sichuan Good Doctor Panxi Pharmaceutical Co., LTD., Xichang, China

**Keywords:** *Periplaneta americana* L., gastric diseases, IGF-1/PTEN/Akt signalling, KFX

## Abstract

**Context:** Kangfuxin (KFX) is widely used for the treatment of gastric and duodenal ulcer; however, more research is needed to determine the protective mechanisms of KFX in ameliorating gastric ulcer.

**Objective:** To investigate the efficacy and potential mechanism of Kangfuxin liquid (KFX) in water-immersion and restraint stress (WIRS)-induced gastric ulcer.

**Materials and methods:** Seventy rats were randomly divided into seven groups (*n* = 10) as follows: the control group (normal saline, i.g.), the model group (normal saline, i.g.), the KFX groups (2.5, 5 and 10 mL/kg, i.g.), the omeprazole group (20 mg/kg, i.p.) and Sanjiuweitai Granules group (1850 mg/kg, i.g.). The WIRS model was applied to induce stress ulcers after 7 days of drug administration. Afterwards, rats were sacrificed at 10 h induced by WIRS.

**Results:** Pre-treatment with KFX (5,10 mL/kg) could effectively reduce the area of gastric ulcers and improve the pathological changes of ulcerated tissue. Moreover, KFX (5,10 mL/kg) increased the prostaglandin E2 (52%) and cyclooxygenase-1 (30%) levels, and improved malondialdehyde (54%), superoxide dismutase (58%), catalase (39%), and nitric oxide (11%) and TNF-α (9%), IL-6 (11%), MMP-9 (54%) and MMP-2 (53%) of ulcer tissue. Furthermore, pre-treatment with KFX dramatically increased IGF-1, PTEN, and Akt protein expression.

**Conclusions:** Our results suggest that KFX has protective effects on WIRS-induced gastric ulcer via inflammatory reactions, oxidative stress inhibition, and pro-survival action, which were the results of activating the IGF-1/PTEN/Akt signalling pathway. Our results provide evidence of KFX for treating gastric ulcer.

## Introduction

Although the concept that a healthy stomach is the gateway to a healthy host has been put forward, gastric ulcers are still one of the most common digestive illnesses worldwide (Hung et al. 2019). Multiple factors are able to induce gastric ulcer, including *Helicobacter pylori* infection, excessive drinking, the use of non-steroidal anti-inflammatory drugs (NSAIDs), and stress (Hunt et al. 2015). Stress-induced gastric ulcer often happens in patients due to major stressful events, such as trauma, shock, and burns. The pathological basis for the development of stress-induced gastric ulcer is multifactorial and includes inflammation and oxidative stress (Cheng et al. 2017; Perez et al. 2017), reduced gastric prostaglandin synthesis, and inhibition of mucosal growth and proliferation. It has been demonstrated that Akt signalling is involved in diverse diseases mainly by regulatory phosphorylation events on Akt (Manning and Toker 2017), which subsequently mediates cell antioxidation (Manning et al. 2019), anti-inflammation (Zhuo et al. 2019) and pro-survival (Ji et al. 2018). Recently, Akt inhibition could suppress gastric cancer cell metastasis has been confirmed (Wang et al. 2016). Moreover, Akt is modulated by many upstream signals, IGF-1 is one of them (Liu et al. 2015) and IGF-1/PTEN/Akt/FoxO signalling pathway is helpful against water immersion and restraint stress-induced gastric ulcers in rats (Huang et al. 2012). Thus, inhibiting oxidation, inflammation and pro-survival may be a target for stress-induced gastric ulcer.

Kangfuxin (KFX), an extract of *Periplaneta americana* L. (Blattidae), has been approved by the National Medical Products Administration (Z51021834), which contains mainly nucleotides, amino acids and small molecular peptides (Lv et al. 2017). KFX is widely used for tissue wound healing, especially in gastric and duodenal ulcers (Chen et al. 2016). Mounting literature has shown that KFX exerts gastroprotective effects by attenuating oxidative stress and endoplasmic reticulum stress against ethanol-induced gastric ulcer in mice (Chen et al. 2016; Shen et al. 2017) and exhibits anticancer activity (Ma et al. 2018). In addition, KFX ameliorates dextran sulphate sodium-induced ulcerative colitis in rats by Keap1/Nrf-2 activation, intestinal barrier function, and gut microbiota regulation. Clinical studies also demonstrated that KFX shows therapeutic effects on chemo/radiotherapy-induced mucositis in nasopharyngeal carcinoma (Luo et al. 2016).

Taken together, the above studies have shown that KFX is a promising therapeutic drug for the treatment of gastric ulcer. Nevertheless, to date, the protective mechanisms of KFX in ameliorating gastric ulcer are still unclear. Based on previous reports, we speculate that KFX improves stress-induced gastric ulcer by inhibiting oxidative stress as well as inflammation and pro-survival. In this study, we utilized water-immersion and restraint stress-induced rat gastric ulcer to investigate the protective effects and mechanism of KFX.

## Materials and methods

### Kangfuxin oral liquid

KFX (Lot. No. 150925), a gift from GoodDoctor Pharmaceutical Group Co., Ltd. (Chengdu, China), which contains 1 g of the dried whole body of *Periplaneta americana* per milliliter according to our previous study (Shen et al. 2017).

### Reagents and antibodies

MDA, SOD, CAT, GSH-Px, NO, PGE2, MMP-2 and TNF-α kits were obtained from Nanjing Jiancheng Bioengineering Institute (Nanjing, China). Anti-COX-1, COX-2, IGF-1, PTEN, p-Akt, Akt, FoxO1, IL-6, TNF-α, MMP-9, α-tubulin primary antibody, and appropriate secondary antibodies were purchased from Santa Cruz Biotechnology (Santa Cruz, CA). Omeprazole was ordered from Yuekang Pharmaceutical Group Co., Ltd. (Beijing, China) and Sanjiuweitai Granules were obtained from China Resources Sanjiu Medical & Pharmaceutical Co., Ltd. (Shenzhen, China). All other reagents were from Sigma-Aldrich (St. Louis, MO).

### Animal treatment

This research was administrated and approved by the Ethics Committee of Peking Union Medical College (Beijing, China). Seventy male Sprague Dawley (SD) rats (6 weeks old, 180–220 g) were obtained from Beijing Vital River Laboratory Animal Technology Co., Ltd. (Beijing, China). Rats were housed in environmentally controlled conditions (22 ± 2 °C, relative humidity of 50 ± 5%) with a 12 h light/dark cycle and had free access to food and water. SD rats were randomly divided into seven groups (*n* = 10): (1) Control group (normal saline); (2) Model group (normal saline); (3)-(5) KFX group (2.5, 5 and 10 mL/kg/day, i.g.); (6) Sanjiuweitai Granules group (SJWT, 1850 mg/kg/day, i.g., positive control); (7) Omeprazole (OME, 20 mg/kg/day, i.p., positive control) group. All rats were pre-treated with listed drugs, respectively, for seven consecutive days.

### Water immersion and restraint stress (WIRS) gastric ulcer

All rats were fasted for 24 h with free access to drinking water before modelling. Each rat was restrained individually in a plastic cage and immersed up to its xiphoid in temperature-controlled water (23 °C) for 10 h. Subsequently, animals were anaesthetized and serum was collected for follow-up analyses. Five stomachs in each group were then ligated at pylorus and cardia and fixed by paraformaldehyde for 24 h. Then, stomach was opened along the greater curvature and rinsed with cold saline to remove the gastric contents. The flattened stomach samples were photographed and the ulcer area (mm^2^) was measured using Image J software. The inhibition percentage was calculated according to the formula: [(Ulcer Area (Vehicle)-Ulcer Area (Treated))/Ulcer Area (Vehicle)] × 100%.

### Histological analysis

The stomach samples of each rat were fixed in 4% buffered paraformaldehyde and embedded in paraffin. Paraffin sections were then cut to a thickness of 5 microns and stained with haematoxylin and eosin (H&E) for histological evaluation according to standard procedures. A microscopic score was measured based on a previous study (Diniz et al. 2015). Each section was determined for (i) disruption of the superficial region of the gastric gland with epithelial cell loss; (ii) interstitial oedema (iii) haemorrhagic damage, using a scale (0: none; 1: mild; 2: moderate; 3: severe) for each criterion.

For immunohistochemical analysis, paraffin sections were deparaffinized with xylene and stained to detect the expressions of cyclooxygenase-1 (COX-1) and COX-2. Afterwards, the stained sections were scanned by the TissueFAXS microscope (TissueFAX plus; TissueGnostics, Vienna, Austria) and counted by using the HistoQuest software (TissueGnostics) as our previous description (Luo et al. 2017).

### Measurement of malondialdehyde, superoxide dismutase, catalase and glutathion peroxidase (MDA, SOD, CAT and GSH-Px) activities

Stomach tissues were homogenized in Tris-buffer on ice and then were centrifuged at 12,000 *g*, 4 °C for 10 min. The supernatants were used to determine the activities of CAT, SOD, GSH-Px and MDA. The concentration of protein in the supernatants was measured by the bicinchoninic acid (BCA) method. The activities of the above four enzymes were determined using the commercial assay kits according to the manufacturer’s instructions, respectively.

### Cytokines evaluation

The levels of cytokines in the serum were evaluated using commercial enzyme-linked immunosorbent assay (ELISA) kits (R&D Systems Inc. Minneapolis, MN) according to the manufacturer’s instruction. The absorbance was read at 450 nm with a microplate reader (Tecan, Switzerland).

### Quantitative real-time PCR

TNF-α, IL-6 and MMP-9 mRNA expression levels were measured according to our previous study (Luo et al. 2015). Briefly, total RNA was isolated from frozen gastric walls samples. cDNA was then synthesized from the isolated RNA. Cycle time values were obtained by conducting real-time PCR with Power SYBR Green PCR Master Mix (Applied Biosystems, Foster City, CA) in an iQ5 Real-time PCR detection system and analysis software (Bio-Rad, Hercules, CA). Relative differences in expression levels were determined using the 2^−ΔΔCT^ method relative to GAPDH gene. Primers (Table 1) were designed using premier primer Software 6.0 (Canadian Premier Life Insurance Company, Toronto, Canada).

**Table 1.** RT-PCR primers for analysis.

Gene	Chain	Sequence
GAPDH	Forward	TTCCTACCCCCAATGTATCCG
	Reverse	CCACCCTGTTGCTGTAGCCATA
IL-6	Forward	GATTGTATGAACAGCGATGATGC
	Reverse	AGAAACGGAACTCCAGAAGACC
TNF-α	Forward	TGAACTTCGGGGTGATCGGT
	Reverse	GGCTACGGGCTTGTCACTCG
MMP-9	Forward	CCCTACTGCTGGTCCTTC
	Reverse	TTGGCTTCCTCCGTGATT

### Western blot analysis

Gastric tissues were lysed in an ice bath with lysis buffer containing 1% proteinase inhibitor cocktail. Protein concentration was determined using a BCA kit with BSA as a standard sample. Western blot assay was then performed according to previous methods. Immunoblots were developed using an ECL kit. Band intensities were analysed using the Gel Pro software (Media Cybernetics, Rockville, MD).

### Statistical analysis

Data were expressed as mean ± SD and analysed by one-way ANOVA followed by Tukey’s multiple comparison test as appropriate (GraphPad Prism version 6 software). *p* value <0.05 was considered significant.

## Results

### KFX protected gastric injury induced by WIRS

**Figure 1.**
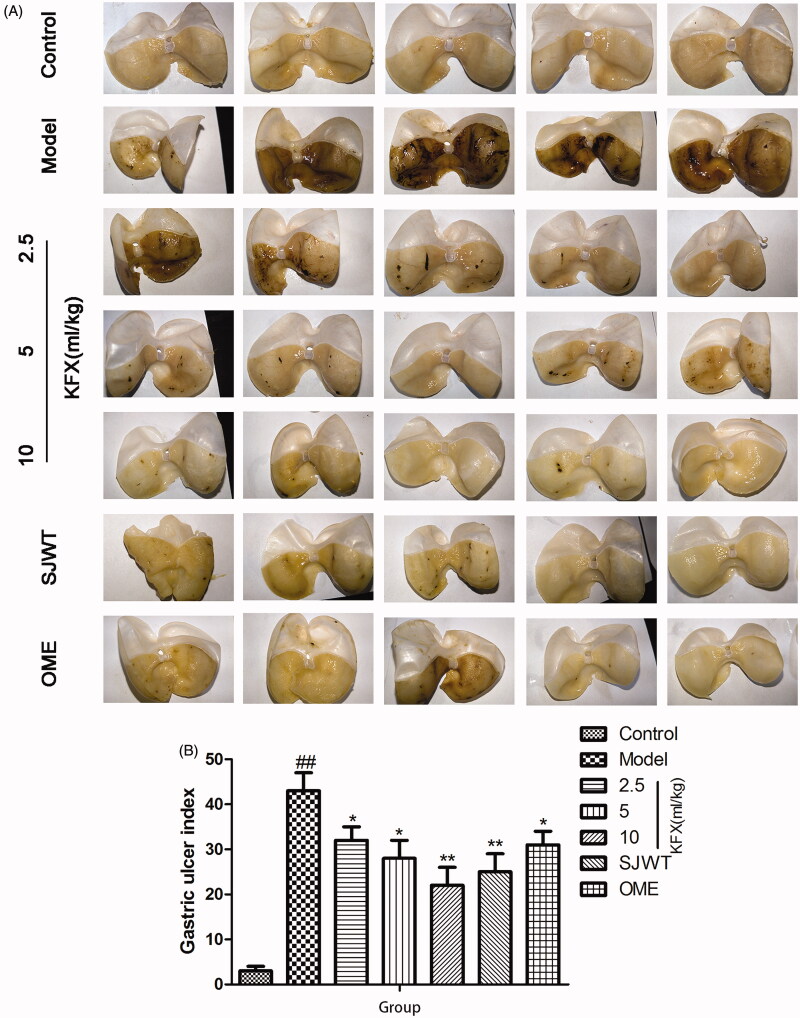
Effects of KFX on the macroscopic morphology and gastric ulcer area of the gastric mucosa in WIRS-treated rats. The data were expressed as the means ± SD (*n* = 5). ^##^*p* < 0.01, compared with the control group; *p < 0.05, **p < 0.01 compared with the model group. KFX: Kangfuxin liquid; SJWT: Sanjiuweitai Granules; OME: Omeprazole.

As shown in Figure 1(A,B), treatment with WIRS remarkably induced gastric diffuse oedema and mucosal damage, resulting in significant higher ulcer index in rats. Pre-treatment with KFX resulted in significant reduction of the lesion area in rats compared with the model group and even better than positive drugs SJWT or OME groups.

### KFX decreased histopathological features in the ulcerated rats

**Figure 2.**
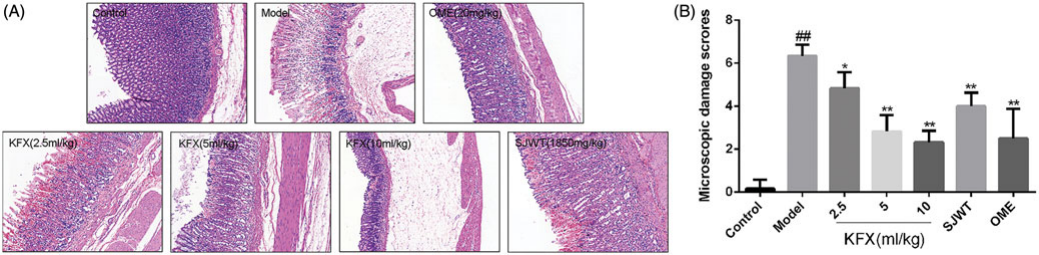
Histological assessment of the effects of KFX on the acute gastric mucosal injuries in WIRS-challenged rats. (A) The gastric tissue was fixed with 4% paraformaldehyde and sectioned to prepare for HE staining. The gastric tissue sections were routinely dehydrated, and then stained with haematoxylin and eosin followed by observation with a microscope. (B) The statistic results of gastric microscopic damage scores. The data were expressed as the means ± SD (n = 5). KFX: Kangfuxin liquid; SJWT: Sanjiuweitai Granules; OME: Omeprazole.

Results of histopathological analysis of the gastric mucosa are demonstrated. In the model group, rats showed severe injury to the gastric epithelium and oedema of submucosa. Whereas, rats pre-treated with KFX (5 mL/kg) improved these alternations, and showed less mucosal damage and milder oedema when compared with the vehicle group (Figure 2(A,B)). Pre-treated with KFX (10 mL/kg) showed normal histology or only very superficial lesions, which was essentially the same as the positive drug OME.

### KFX-regulated activity of oxidase in the damaged gastric tissue

**Figure 3.**
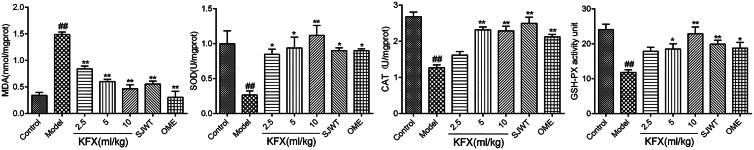
The effect of KFX pre-treatment on MDA, SOD, CAT, GSH-Px activities in the gastric tissues of WIRS-induced rat ulcer model. The data were expressed as the means ± SD (*n* = 5). ^##^*p* < 0.01, compared with the control group; *p < 0.05, **p < 0.01 compared with the model group. KFX: Kangfuxin liquid; SJWT: Sanjiuweitai Granules; OME: Omeprazole.

According to the results (Figure 3), WIRS caused a remarkable increasing MDA level compared to control rats. Treatment with KFX led to significant decrease in gastric MDA relative to model group rats. On the contrary, GSH, CAT and SOD levels were greatly decreased by WIRS compared to control group. KFX pre-treatment significantly increased the gastric GSH and SOD levels compared to model group rats.

### KFX modulated inflammatory factors expression in the damaged gastric tissue

**Figure 4.**
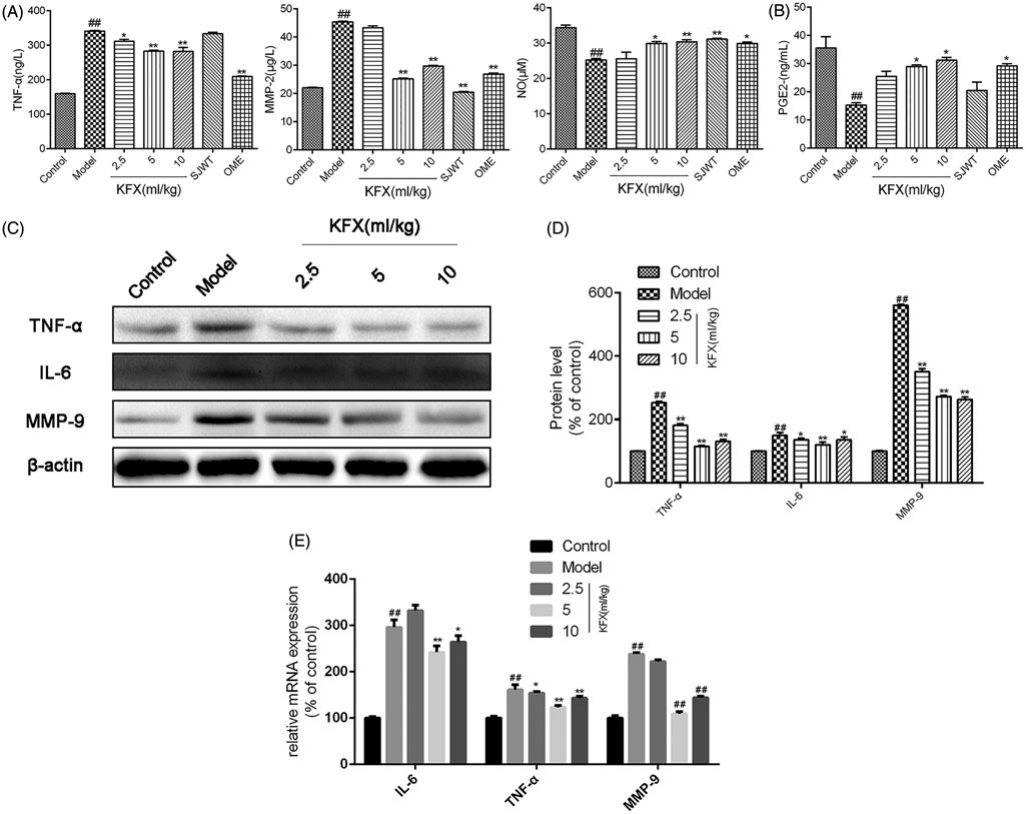
KFX modulated inflammatory factors expression in WIRS-induced gastric ulcer rats. (A and B) The gastric tissue homogenate was prepared to determine the levels of TNF-α, MMP-2, PGE2 and NO by ELISA. (C) The gastric samples were prepared with lysis buffer, resolved on 10% SDS-PAGE, and transferred to a PVDF membrane. Western blots were performed to detect the total or MMP-9, IL-6 and using specific primary antibodies. (D) Densitometric analysis was used to quantify the levels of TNF-α, IL-6 and MMP-9. (E) The total RNA in gastric tissues was extracted to detect the COX-1, COX-2, IL-6, TNF-α, MMP-9, and GAPDH mRNAs by QPCR. The data were expressed as the means ± SD (n = 5). ^##^*p* < 0.01, compared with the control group; *p < 0.05, **p < 0.01 compared with the model group. KFX: Kangfuxin liquid; SJWT: Sanjiuweitai Granules; OME: Omeprazole.

The tissue homogenate levels of pro-inflammatory cytokines TNF-α and MMP-2, and anti-inflammatory cytokines NO and PGE2 were measured by ELISA assay. As indicated in Figure 4(A,B), KFX pre-treatment ameliorated the elevated levels of TNF-α and MMP-2 and restored the depleted NO and PGE2 level compared to the model group. To further detect the anti-inflammatory effects of KFX, we measured TNF-α, IL-6, MMP-9 protein expression. As shown in Figure 4(C,D), KFX sharply reduced these inflammatory factors expression, which was also demonstrated at the gene level (Figure 4(E)).

### KFX increased COX-1 expression in the damaged gastric tissue

**Figure 5.**
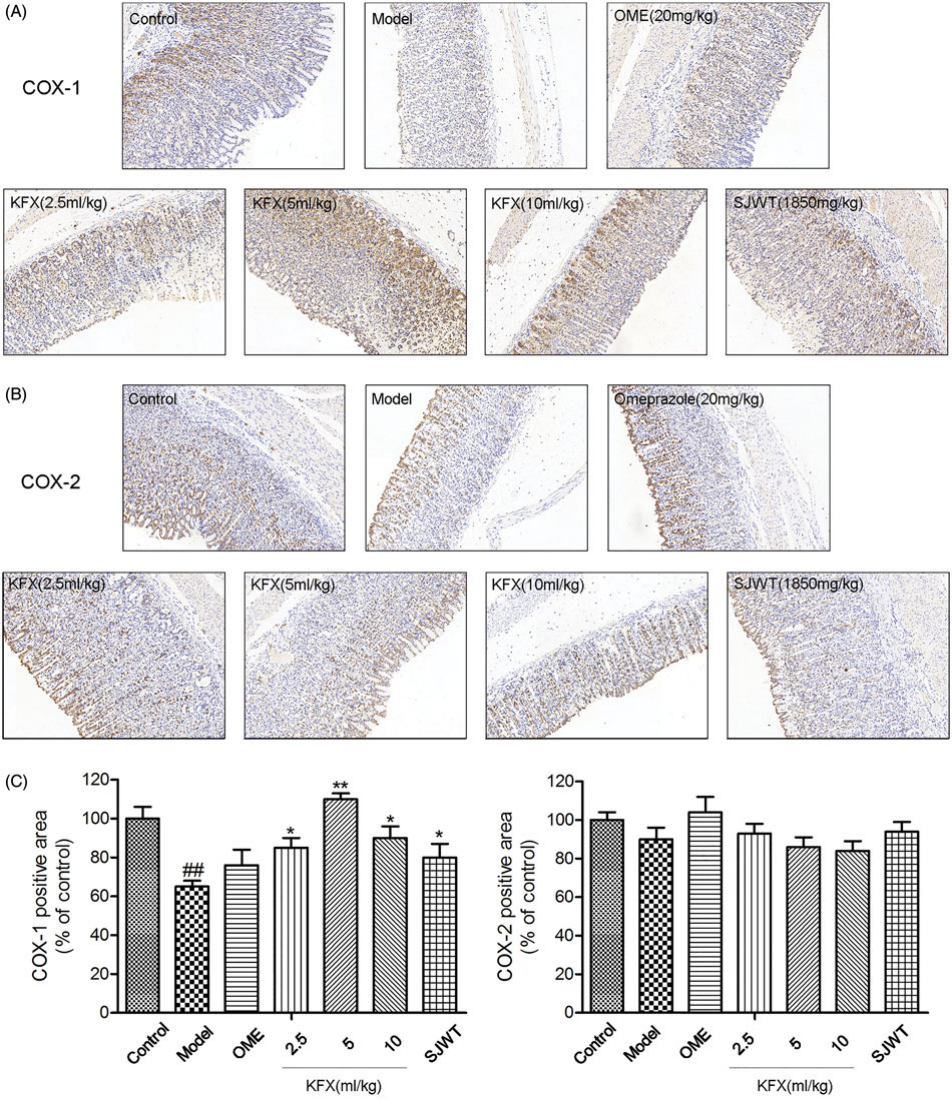
Effects of KFX on COX-1 and COX-2 expression levels in stomach tissue of WIRS-treated rats. (A, B) Photomicrographs of immunohistochemical staining for determination of COX-1 and COX-2 protein expression in gastric mucosa. (C) Quantitative assessment of COX-1 and COX-2 protein expression. The data were expressed as the means ± SD (*n* = 5). ^##^*p* < 0.01, compared with the control group; *p < 0.05, **p < 0.01 compared with the model group. KFX: Kangfuxin liquid; SJWT: Sanjiuweitai Granules; OME: Omeprazole.

To assess the prostaglandin production limiting enzyme expression in rat gastric ulcers, sections were stained with COX-1 and COX-2 antibodies. As demonstrated in Figure 5(A,C), COX-1 expression level was obviously lower compared with control group. Pre-treatment with KFX could significantly reverse the decreased COX-1 level. However, there was no significant difference in various groups in terms of COX-2 expression level.

### KFX activated IGF-1/akt pathway in WIRS-induced ulcer rats

**Figure 6.**
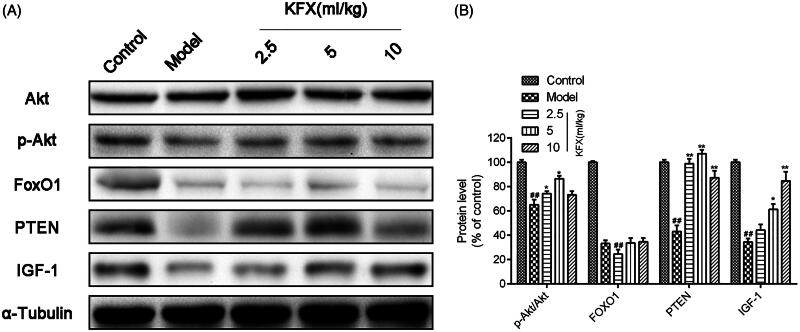
KFX activated IGF-1/Akt pathway in WIRS-induced ulcer rats. (A) The gastric samples were prepared with lysis buffer, resolved on 10% SDS-PAGE, and transferred to a PVDF membrane. Western blots were performed to detect the total or Akt, p-Akt, FoxO1, PTEN, IGF-1 using specific primary antibodies. (B) Densitometric analysis was used to quantify the levels of p-Akt/Akt, FoxO1, PTEN, IGF-1. The data were expressed as the means ± SD (*n* = 5). ^##^*p* < 0.01, compared with the control group; *p < 0.05, **p < 0.01 compared with the model group. KFX: Kangfuxin liquid.

To determine the protective mechanism of KFX against WIRS-induced ulcer rats, we tested the IGF-1/Akt signalling related protein expression. As indicated in Figure 6(A,B), we found KFX could significantly increase p-Akt/Akt, PTEN and IGF-1 expression levels, which was consistent with the previous study (Huang et al. 2012). However, KFX has no obvious effect on FoxO1 expression.

## Discussion

Although our group has previously confirmed the gastroprotective effects of KFX in ethanol-induced gastric ulcer and gastric cancer (Chen et al. 2017; Shen et al. 2017), little is known about its potential therapeutic effects on stress-induced gastric ulcer. At present, the primary animal models used for model systems are prepared on the basis of humans’ way of life and ulcer inducers, such as ethanol (Aziz et al. 2019), NSAIDs (Piao et al. 2018) and WIRS (Nur Azlina et al. 2017). It is well known that WIRS-induced gastric injury major on gastric mucosa, resulting in gastric mucosa erosion, oxidative stress and high inflammatory factors expression (Fu et al. 2018).

In the present study, we first detected the antiulcer effect of the KFX, using a conventional test method. Later, its efficacy was demonstrated to be similar to that of omeprazole based on the ulcer index result. The WIRS induced a series of pathological alterations and lesions in the stomach, and gastric ulcer is a process that results from multiple factors. Then, we measured the histological changes of gastric mucosa. Our results demonstrated that KFX pre-treatment could significantly improve the gastric mucosal haemorrhage compared with model group based on the microscopic scores. Oxidative and inflammatory injury is a crucial event in gastric ulcer formation (Aziz et al. 2019). To further confirm our conclusion, we determined the oxidative enzymes and inflammatory factor expression in gastric mucosa. Consistently, our results showed that KFX could remarkably increase antioxidative enzymes expression level, and reduce inflammatory factor expression. Defensive factors, NO and PGE2, play a pivotal role in gastric mucosal function (Wang et al. 2018). Expectedly, pre-treatment with KFX was able to increase NO and PGE2 release. We next measured COX-1 and COX-2 expression, which is the upstream of PGE2 (Shu et al. 2019). As shown in Figure 5, KFX could notably increase COX-1 expression and is superior to OME. Interestingly, KFX has no effect on COX-2 expression. These results indicated that KFX has preferential COX-1 stimulating activity.

The aforementioned results arouse our interest to investigate the underlying mechanism of KFX, which was still not thoroughly understood. Akt signalling activation is a typical pathway involved in cell proliferation (Chu et al. 2018). And it is regulated by multiple upstream proteins, such as PI3K (Luo et al. 2014) and PTEN (Lee et al. 2013). Previous literature has reported that the IGF-1/PTEN/Akt/FoxO signalling pathway plays a certain role in the protection against ulceration through the regulation of cellular apoptosis as observed in the development and healing of rat gastric ulcers (Huang et al. 2012), which strengthen our research on this signal pathway. As expected, KFX pre-treatment could significantly increase Akt phosphorylation, PTEN and IGF-1 expression level. However, it has no distinct effect on FoxO1 expression.

Taken together, this study demonstrated the protective effects of KFX in rat models of water immersion restraint stress-induced gastric mucosal damage and revealed antioxidative and anti-inflammatory mechanisms of KFX based on the activation of IGF/PTEN/Akt mediated signalling pathways for this effect. Our results provide scientific evidence to support the beneficial use of KFX to treat gastric ulcers. However, further studies to elucidate the exact targets of KFX should be conducted to obtain additional information that would potentially be useful for future drug clinical application.
